# A role of point-of-care ultrasound in the emergency department diagnosis of vision loss due to traumatic cataract

**DOI:** 10.1186/s12245-023-00558-1

**Published:** 2023-11-02

**Authors:** Christian A. Tagle, Joe W. Chen, Jamshid Mistry, Danny Fernandez, Cameron C. Neeki, Fanglong Dong, Michael M. Neeki

**Affiliations:** 1 Department of Internal Medicine, Arrowhead Regional Medical Center, Colton, CA USA; 2 California University of Science and Medicine, Colton, CA USA; 3 Department of Emergency Medicine, Arrowhead Regional Medical Center, 400 N. Pepper Ave, Suite # 107, Colton, CA USA

**Keywords:** Vision loss, Cataract, Emergency department, Point-of-care, Ultrasound

## Abstract

**Background:**

Ocular complaints, including acute or subacute vision loss, are commonly encountered in emergency departments (ED). These potentially time-sensitive complaints are difficult to diagnose and evaluate without adequate, specialized equipment and expertise. Additionally, a thorough evaluation often requires a more extensive and specialized physical exam, imaging, and ophthalmologic consultation, all of which may not be readily available in the acute setting.

**Case presentation:**

This case report presented a patient in the emergency department with the chief complaint of vision loss. Point-of-care ultrasound (POCUS) using the 10-MHz-linear-array probe, in the ocular setting, demonstrated calcification of the lens, a finding consistent with cataract in the right eye.

**Conclusions:**

The use of POCUS can expedite the accurate identification of vision threatening pathology, such as cataracts, and streamline ED disposition and plan of care.

## Background

Ocular complaints, specifically acute vision loss, are commonly encountered in the emergency department (ED) [1]. Typically, time-sensitive pathologies are difficult to diagnose without adequate, specialized equipment and expertise readily available [2]. Additionally, thorough evaluation often requires a more extensive and specialized physical exam, imaging, and consultation with a specialist [3, 4]. The recent advent in use of ocular point of care ultrasound (POCUS) in the ED has shown to be of great clinical utility when attempting to diagnose various ocular pathologies that are both acute and chronic in nature [1, 2, 5–7]. Many uses of ocular POCUS in the ED have been described, and some of which have developed well-established clinical protocols, especially with regard to pathologies often seen with posterior chamber abnormalities. These include retinal detachment, vitreous hemorrhage, retinal artery occlusions, traumatic retrobulbar hematoma, and others. However, the use of ocular POCUS to evaluate suspected cases of cataracts has not been well-reported in the literature [1, 2, 5–9]. One retrospective study that evaluated a cataract detection and treatment campaign found that in the evaluation of medium to severe opacities of the lens of the eye, 77.5% of eyes examined revealed evidence of vitreous detachment, a finding that could explain the initial presenting symptom of poor vision [10]. It should be noted that cataract could be due to aging or ocular trauma, POCUS allowed for the assessment of these opacities in a manner that would otherwise require the expertise of a specialist. Additionally, POCUS can also simultaneously assess for other ocular pathologies, such as retinal detachment and vitreous hemorrhage. In a similar fashion, we find value in describing the utility of POCUS by examining the case of an elderly male who presented to a regional ED with a chief complaint of subacute, right-sided vision loss. The specific findings noted on the POCUS exam helped the ED physician’s discussion with the consulting ophthalmologist to expedite further management and disposition planning.

### Case presentation

**Fig. 1:**
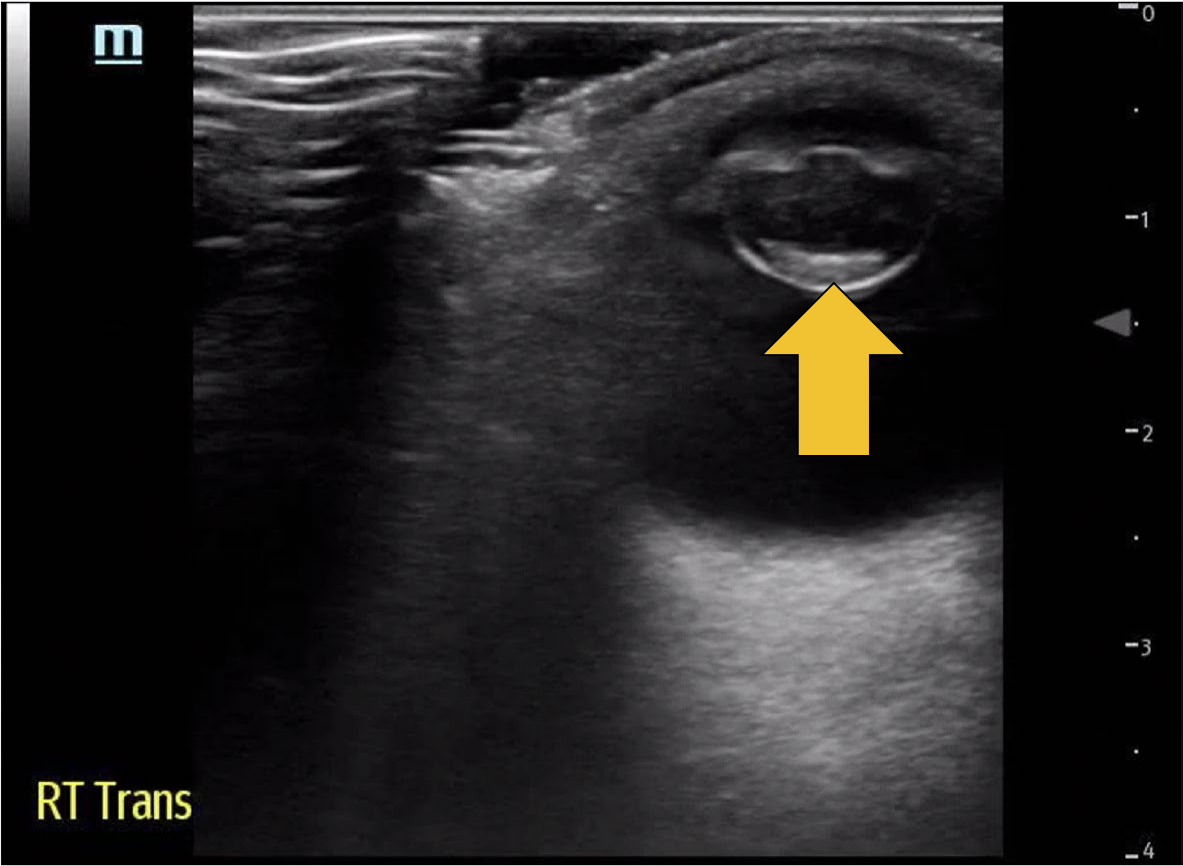
Transverse view of the right globe demonstrating a hyperechoic density, as seen at the arrow tip, consistent with a cataract

A 67-year-old Hispanic male with no reported past medical history presented to the ED with the chief complaint of 3 months of right eye vision loss. The patient stated his vision loss started after a blade of grass was caught in his eye, prompting him to rub the affected eye. He reported the initial itching sensation improved; however, his vision loss continued to deteriorate. He denied eye pain, discharge, floaters, flashers, or diplopia. In the ED, physical examination was notable for opacification of the right lens, visual acuity of 20/40 on the left, and only light perception on the right, with no blink to threat appreciated. POCUS using the 10-MHz-linear-array probe, in the ocular setting, demonstrated calcification of the lens, a finding consistent with cataract in the right eye (Fig. 1). Consensual pupillary light response of the right eye was also visualized using the ultrasound probe. Importantly, there was no evidence of retinal detachment or vitreous hemorrhage and the optic nerve measurements were within normal range. Anterior segment testing using slit-lamp was performed without any cells in flare. Furthermore, no fluorescein uptake was noted, and eye pressures were within normal range.

Ophthalmology was consulted, and the case was reviewed; they believed that given the patient’s history, clinical presentation, physical examination, and ultrasound findings, symptoms were most likely secondary to a mature cataract of the right eye. Outpatient ophthalmology follow-up for formal diagnosis, possible cataract extraction, and intraocular lens implantation was recommended. Follow-up communication with the patient revealed that he had been evaluated and received his formal diagnosis from the ophthalmologist in the outpatient setting.

## Discussion and conclusions

A cataract is a partial or total opacification of the crystalline lens of the eye, and it is the most common cause of visual impairment worldwide [11]. In adults, cataracts are most commonly age-acquired, with presenile cataracts presenting around age 45–55 years and senile cataracts manifesting after this range [11, 12]. Age-related cataracts have been estimated to affect 17 million people worldwide with blindness, contributing to almost half of all causes of blindness [4, 13–15]. However, in the assessment of acute or chronic vision loss, a number of other potential causes must first be ruled out as they may be iatrogenic, associated with other ocular or systemic disease, inflammatory, or induced by ocular trauma [14, 16, 17].

For any patient who presents to the ED with complaints of visual disturbances, examination should include, but is not limited to, external exam, visual acuity testing, visual field testing, pupillary exam, and extra-ocular movements. Additionally, slit-lamp exam, ophthalmoscopy, and intraocular pressure testing should be performed [7].

Although cataracts are commonly seen in the ED, a formal diagnosis is traditionally given after thorough evaluation by an ophthalmologist [2]. With regard to symptomatology, it is dependent on the location of the cataract (nuclear vs cortical vs posterior subcapsular), but may include reports of glare, such as halos around lights, particularly at night, requiring more light to improve the ability to see well. Untreated, the patient will eventually complain of a painless blurring of vision [11]. Other symptoms such as photophobia, monocular diplopia, and myopic shift, may be found depending on the anatomical distribution of the opacities [11].

Diagnosis is often based on slit-lamp examination after pupillary dilation; however, there has been a growing amount of research documenting the clinical utility of ocular ultrasound in identifying ocular pathology in patients with acute vision changes or eye trauma [2, 5, 7, 8]. Examples of such pathology include retinal and vitreous detachments, central retinal artery occlusion, vitreous hemorrhage, ectopia lentis, papilledema, optic neuritis, and globe rupture [2, 8]. POCUS provides a quick, accurate, and well-tolerated, noninvasive tool for evaluation of vision threatening pathology [1, 2, 8]. These findings, in conjunction with specialist consultation, allowed for a more rapid and streamlined disposition and plan of care [8].

In the case of our patient who had described an inability to see out of his right eye for 3 months, rapid bedside POCUS of his globe provided a quick and accurate diagnosis of cataracts with a possible etiologic relation to trauma, consistent with both his subjective report of present illness and our objective physical exam findings. Additionally, POCUS also simultaneously assessed for other ocular pathologies, such as retinal detachment and vitreous hemorrhage. Ultimately, following discussion with ophthalmology, the decision was made to discharge the patient home with instructions to follow up in the outpatient setting for further management of his cataract.

## Conclusions

Surmounting evidence in recent literature documents that emergency medicine practitioners can use POCUS to accurately identify vision threatening pathology such as retinal or vitreous detachments, vitreous hemorrhage, and as seen in our case, cataract formation. Of note, ocular ultrasound is not intended to replace the role of the ophthalmologist for definitive diagnosis of these conditions, but it may serve as an adjunct to help emergency medicine practitioners improve care for patients with ocular symptoms.

## Data Availability

The datasets used and/or analyzed during the current study are available from the corresponding author on reasonable request.

## Abbreviations

ED: Emergency department
POCUS: Point-of-care ultrasound

## References

[1] Blaivas M, Theodoro D, Sierzenski PR (2002). A study of bedside ocular ultrasonography in the emergency department. Acad Emerg Med.

[2] Skidmore C, Saurey T, Ferre RM, Rodriguez-Brizuela R, Spaulding J, Mason NL (2021). A narrative review of common uses of ophthalmic ultrasound in emergency medicine. J Emerg Med.

[3] Delbarre M, Froussart-Maille F (2020). Signs, symptoms, and clinical forms of cataract in adults. J Fr Ophtalmol.

[4] Francis PJ, Berry V, Moore AT, Bhattacharya S (1999). Lens biology: development and human cataractogenesis. Trends Genet.

[5] Propst SL, Kirschner JM, Strachan CC, Roumpf SK, Menard LM, Sarmiento EJ (2020). Ocular point-of-care ultrasonography to diagnose posterior chamber abnormalities: a systematic review and meta-analysis. JAMA Network Open.

[6] Lin C-H, Ling XC, Wu W-C, Chen K-J, Hsieh C-H, Liao C-H (2021). The Role of Nonophthalmologists in the Primary Evaluation of Head Injury Patients with Ocular Injuries. J Pers Med.

[7] Juang PS, Rosen P (1997). Ocular examination techniques for the emergency department. J Emerg Med.

[8] Dornhofer K, Alkhattabi M, Lahham S (2020). Point-of-care ultrasound detection of cataract in a patient with vision loss: a case report. Clin Pract Cases Emerg Med.

[9] West SK, Taylor HR (1986). The detection and grading of cataract: an epidemiologic perspective. Surv Ophthalmol.

[10] Mendes MH, Betinjane AJ, Cavalcante AdS, Cheng CT, Kara-José N (2009). Ultrasonographic findings in patients examined in cataract detection-andtreatment campaigns: a retrospective study. Clinics.

[11] Thompson J, Lakhani N (2015). Cataracts. Prim Care.

[12] Tan AC, Wang JJ, Lamoureux EL, Wong W, Mitchell P, Li J (2011). Cataract prevalence varies substantially with assessment systems: comparison of clinical and photographic grading in a population-based study. Ophthalmic Epidemiol.

[13] Dotsenko V, Neshkova E, Namazova I, Yarovaya G (1996). Hageman factor and kallikrein in pathogenesis of senile cataracts and the pseudoexfoliation syndrome. Immunopharmacology.

[14] Van Heyningen R (1976). Sugar alcohols in the pathogenesis of galactose and diabetic cataracts. Birth Defects Orig Artic Ser.

[15] Meltzer ME, Congdon N, Kymes SM, Yan X, Lansingh VC, Sisay A (2017). Cost and expected visual effect of interventions to improve follow-up after cataract surgery: prospective review of early cataract outcomes and grading (PRECOG) study. JAMA ophthalmology.

[16] McCusker MM, Durrani K, Payette MJ, Suchecki J (2016). An eye on nutrition: The role of vitamins, essential fatty acids, and antioxidants in age-related macular degeneration, dry eye syndrome, and cataract. Clin Dermatol.

[17] Nordmann J (1957). The pathogenesis of cataracts. Bibl Ophthalmol.

